# Incidence and recurrence risk of low birth weight in Northern Tanzania: A registry based study

**DOI:** 10.1371/journal.pone.0215768

**Published:** 2019-04-22

**Authors:** Miriam H. Mvunta, Innocent B. Mboya, Sia E. Msuya, Beatrice John, Joseph Obure, Michael J. Mahande

**Affiliations:** 1 Department of Epidemiology and Biostatistics, Institute of Public Health, Kilimanjaro Christian Medical University College, Moshi, Tanzania; 2 Department of Community Health, Institute of Public Health, Kilimanjaro Christian Medical University College, Moshi, Tanzania; 3 Department of Obstetrics and Gynecology, Kilimanjaro Christian Medical Centre (KCMC), Moshi, Tanzania; National Institute of Health, ITALY

## Abstract

**Background:**

Low birth weight (LBW) is an important indicator of newborn survival. It is associated with higher risk of morbidity, mortality and long-term health consequences. Little has been done on incidence and recurrence risk of LBW in developing countries including Tanzania. This study aimed to determine the incidence and recurrence risk of LBW among women who delivered at Kilimanjaro Christian Medical Center (KCMC), Tanzania.

**Methods:**

A hospital-based prospective cohort study was conducted using maternally-linked data from KCMC birth registry between 2000 and 2010. A total of 26,191 women delivered singleton live babies during the study period. Of these, 4,603 (17.6%) had subsequent pregnancies. The recurrence risk of LBW was estimated using a multivariable log-binomial regression model. A robust variance estimator was used to account for correlation between births of the same mother.

**Results:**

The incidence of LBW was 7.1%. The absolute recurrence risk of LBW was 28.1%. This corresponds to a relative risk (RR) of 5.08-fold, 95% CI 4.01–6.45). Antenatal care visits (<4) (RR: 5.00; 95% CI 3.58–6.98), preterm birth (RR: 4.55; 95% CI 3.21–6.43), positive HIV status (RR: 7.49: 95% CI 3.91–14.36) and preeclampsia (RR: 4.37; 2.60–7.35) in the first pregnancy were important predictors of LBW recurrence.

**Conclusion:**

The incidence of LBW and its recurrence was high in the study population. Women with previous history of LBW had higher risk of recurrent LBW in subsequent pregnancies. Identification of factors associated with LBW recurrence, proper post-partum care management to ensure Healthy Timing and Spacing of Pregnancy, Pre-conception care and close clinical follow-up during subsequent pregnancy may help reduce LBW recurrence.

## Introduction

Low birth weight (LBW) is defined as the newborn weight at birth of less than 2500 grams regardless of gestational age [1–3]. It is an important indicator for newborn survival. LBW may result from prematurity or intra uterine growth retardation or both [1]. It is associated with higher risk of morbidity, mortality and long-term life health consequences [4]. It has been estimated that 15% to 20% of all births worldwide are low birth weight, which corresponds to more than 20 million births a year [3].

Numerous factors have been associated with increased risk of LBW. These include preeclampsia, eclampsia, placental abruption, placenta previa, premature rupture of membrane (PROM), maternal background characteristics such as poor nutrition, smoking during pregnancy, maternal illness during pregnancy, high parity, low maternal education, high maternal age, low maternal Body Mass Index (BMI), inadequate prenatal attendance, medical conditions such as HIV positive status of the mother and maternal anemia, environmental as well as genetic factors like history of mother having been born with LBW and history of LBW in prior pregnancies [2, 4–8].

The risk of LBW has been reported to recur between pregnancies. Women with previous history of LBW have been reported as potential carriers of the recurrent risk and tend to have higher recurrence risk of LBW in their subsequent pregnancy compared to those who had a previous normal birth weight baby [9–14].

A number of factors have been associated with increased risk of recurrent LBW including history of LBW, high parity, both extreme maternal age, smoking during pregnancy, low BMI, HIV positive status, lower socio-economic class, previous history of preterm birth, female sex of the newborn, low quality antenatal care and low maternal weight gain during pregnancy [10–13, 15, 16]. Recurrent LBW has also been associated with intergenerational component, for example, an infant born with LBW is at risk of delivering LBW babies [17]. Little has been documented on the incidence and recurrence risk of LBW in developing countries including Tanzania.

Understanding the recurrence risk of LBW and associated factors may help to design a focused intervention for groups of mothers at high risk of LBW recurrence. This study aimed to determine the incidence and recurrence risk of LBW among women who delivered at Kilimanjaro Christian Medical Center (KCMC), Tanzania.

## Materials and methods

### Study design and setting

A hospital-based prospective cohort study was conducted using maternally-linked data from KCMC birth registry for women who delivered live singletons between 2000 and 2010. KCMC is one of the four referral hospitals in Tanzania which is located in Moshi, Kilimanjaro region in North of Tanzania. The hospital receives women from nearby communities and referrals from the nearby regions such as Arusha, Manyara and Tanga. The hospital has an average of 4,000 deliveries per year. By the end of December 2010, a total of 26,191 women were recorded at birth registry to have been delivered at KCMC. These women contributed to 30,800 siblings.

### Data source and collection

The study utilized KCMC maternally-linked data which are available at the computerized KCMC medical birth registry. The KCMC medical birth registry has been operating since July 2000. The detail of the medical birth registry has been well described elsewhere [18]. All women who gave birth at KCMC are assigned with a unique hospital identification number which is constant for subsequent births that occur at KCMC for the same mother. This enables linkage of each woman with their respective siblings in subsequent births.

Trained midwives collected all information from mothers using a standardized questionnaire within 24 hours of hospital delivery, or as soon as a mother has recovered from birth. Furthermore, additional information was extracted from patient files while mothers are also asked to bring their antenatal cards for more clarification on prenatal information such as antenatal care visits, maternal weight and blood pressure measurements. Finally, all data are entered and stored in a computerized data base system at the birth registry.

### Participants

**Fig 1:**
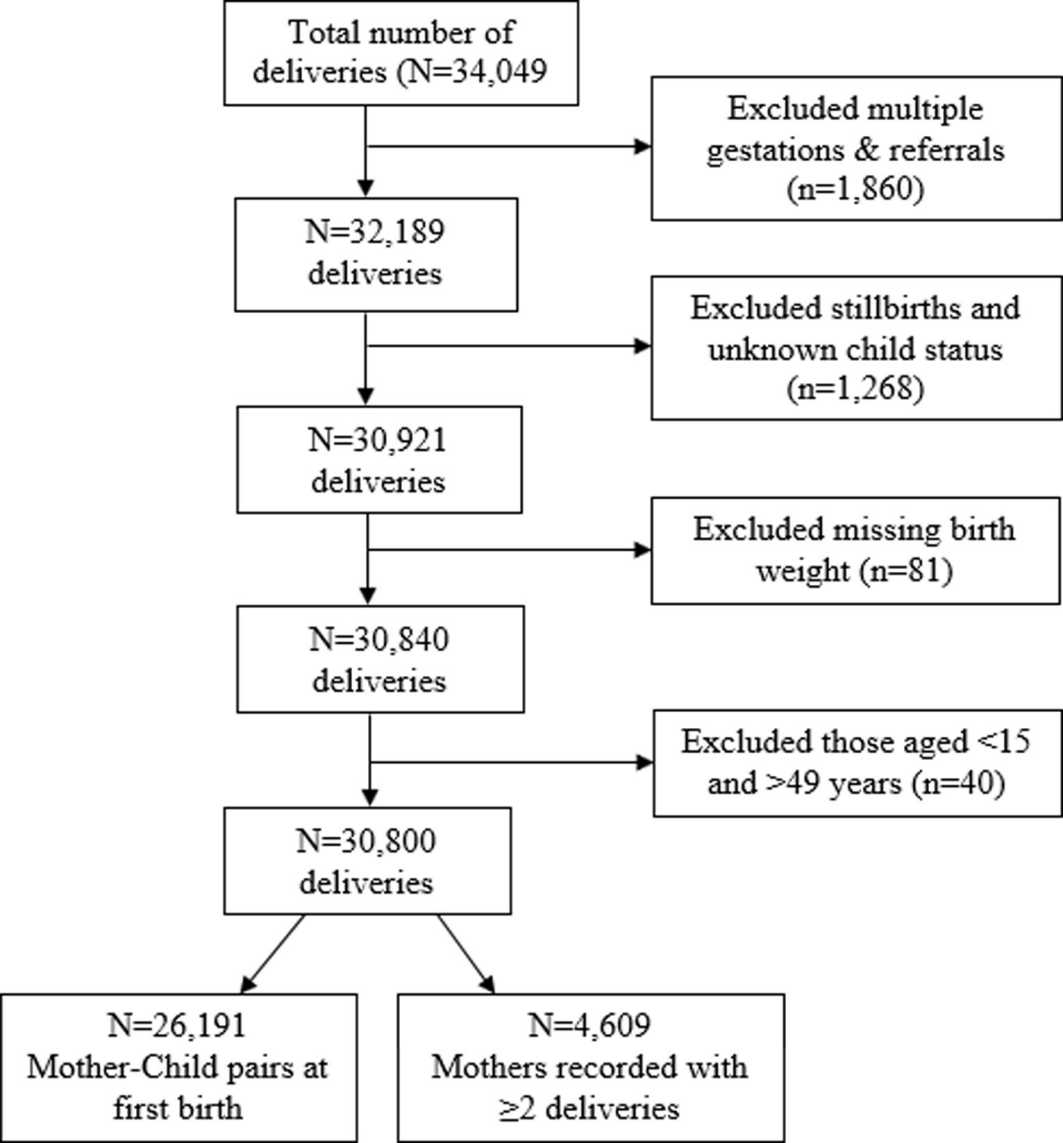
Schematic presentation of the cohort follow-up. Data from the 2000–2010 KCMC medical birth registry.

This study included mothers who had at least single LBW baby followed by a subsequent birth before 2010. The inclusion criteria required all records to have a hospital number to enable linkage of mother with their respective siblings. We excluded all women with multiple gestations, those missing records on number of deliveries (i.e. whether singleton or multiples) and unknown child status (i.e. whether dead or alive), stillbirths and missing birth weight. The remaining 26,191 comprised the final sample size (Fig 1).

### Study variables

The main outcome variable was LBW. LBW was defined as birth weight of less than 2500 grams. Recurrence of LBW was defined as repetition of LBW delivery in a subsequent pregnancy [19]. Independent variables include maternal demographic characteristics: age in years, marital status, education level, area of residence, alcohol consumption during pregnancy, smoking during pregnancy, number of antenatal care (ANC) visits, preterm birth (defined as gestational age at birth <37 weeks), mode of delivery (whether vagina or by cesarean section) and body mass index (BMI). Maternal medical conditions included HIV status, preeclampsia, hypertension, anemia, abruption placenta, and placenta previa. Child characteristics included status after birth (i.e. whether a live born, transferred to intensive care unit (ICU) or neonatal death episode) and sex of the child.

### Statistical analysis

Data were analyzed using STATA version 13.1 (Stata Corp., Texas and US). Numeric variables were summarized using mean (standard deviation) while comparison of means was performed using Student’s t-test. Multivariate log-binomial regression model was used to estimate the relative risk and recurrence risk of LBW with 95% confidence intervals (CIs). A robust variance estimator was used to account for correlation between births of the same mother. A p-value of less than 5% was considered statistically significant.

### Ethical consideration

Ethical approval was obtained from Kilimanjaro Christian Medical University College Research Ethics and Review Committee (CRERC) and the National Institute for Medical Research of Tanzania. Written informed consents were sought previously from all mothers during the interview. Both confidentiality and privacy were ensured where unique mother identification number were used instead of names while interviews were conducted in a separate room.

## Results

There were 26,191 women who delivered at KCMC between 2000–2010, this corresponds to 30,800 deliveries. Out of these, 2,497 (9.5%) women had LBW infants in their first pregnancy. A total of 4,603 women confirmed to have more than one delivery within the follow up time, out of which 325 (7.1%) had recurrent LBW.

### LBW by maternal socio-demographic characteristics at first birth

Socio-demographic characteristics of 26,191 mothers in the first pregnancy are shown in Table 1. Mothers who were aged 15–19 and 35–49 years had slightly higher proportion of infants with LBW as compared to those aged 20–34 years (11% vs 9% respectively). Likewise, the proportion of LBW was also higher among mothers who delivered preterm (<37 weeks of gestation) and those with less than four ANC visits (40.2% and 16.4%) compared to those who delivered at term (≥37 weeks of gestation) and had ≥4 ANC visits (5.8% and 6.5%) respectively.

**Table 1:** Maternal characteristics in the first pregnancy (N = 26,191).

Characteristics	Outcome in first pregnancy	
	Total (N = 26,191)	LBW 2,497 (9.5%)
**Mothers age (years)**		
20–34	20,322 (77.6)	1,853 (9.1)
15–19	2,721 (10.4)	305 (11.2)
35–49	3,148 (12.0)	339 (10.8)
Residence^*^		
Rural	11,316 (43.4)	1,208 (10.7)
Urban	14,785 (56.7)	1,280 (8.7)
Maternal education (years)^*^		
<12 Years	17,953 (68.7)	1,839 (10.2)
≥12 Years	8,189 (31.3)	653 (8.0)
Marital status^*^		
Married	22,884 (87.8)	2,091 (9.1)
Unmarried	3,296 (12.3)	388 (12.1)
Alcohol use during pregnancy^*^		
No	17,210 (65.8)	1,781 (10.4)
Yes	8,932 (34.2)	708 (7.9)
**Gestation age (weeks)** ^*^		
<37	2,469 (10.3)	993 (40.2)
≥37	21,554 (89.7)	1,243 (5.8)
Mode of delivery^*^		
Vaginal	17,681 (68.0)	1,441 (8.2)
Caesarian section	8,318 (32.0)	1,011 (12.2)
Attendance to antenatal care^*^		
<4 visits	7,201 (28.1)	1,182 (16.4)
≥4 visits	18,396 (71.9)	1,201 (6.5)

^*^Frequency and percentages do not tally to the total due to missing values.

### Predictors of LBW in subsequent pregnancy by maternal characteristics in the first pregnancy

In the multivariable analysis mothers who delivered LBW baby in the first pregnancy had over 5 times higher risk of having LBW in subsequent pregnancies (RR: 5.08; 95% CI 4.01–6.45), an absolute recurrence risk of 28.1%. Likewise, mothers who had less than four ANC visits (RR: 1.45; 95% CI 1.09–1.93), delivered a preterm baby in the first pregnancy (RR: 2.67; 95% CI 1.98–3.60), those with HIV positive status (RR: 1.99; 95% CI 1.05–3.78) and those who had preeclampsia (RR: 2.24; 95% CI 1.47–3.42) had significantly higher risk of delivering a LBW baby in subsequent pregnancies (Table 2).

**Table 2:** Predictors of LBW in subsequent pregnancy by maternal characteristics in first pregnancy.

Maternal characteristics	LBW in subsequent pregnancies					
	At risk	n (%)	RR*	95%CI	RR**	95%CI
**LBW in first pregnancy**						
Yes	366	103 (28.1)	5.37	4.36–6.61^†^	5.08	4.01–6.45^†^
No	4,237	222 (5.2)	Ref		Ref	
**Area of residence**						
Rural	1,985	160 (8.1)	1.31	1.06–1.62^††^	1.18	0.93–1.51
Urban	2,587	159 (6.2)	Ref		Ref	
**Attendance to ANC**						
<4	767	75 (9.8)	1.54	1.20–1.97^††^	1.45	1.09–1.93^††^
≥4	3,740	238 (6.4)	Ref		Ref	
**Gestational age (weeks)**						
<37	344	61 (17.7)	2.88	2.23–3.73^†^	2.67	1.98–3.60^†^
≥37	3,965	244 (6.2)	Ref		Ref	
**HIV status**						
Positive	87	12 (13.8)	2.07	1.20–3.59^†^	1.99	1.05–3.78^††^
Negative	2,058	137 (6.7)	Ref		Ref	
**Preeclampsia/eclampsia**						
Yes	175	24 (13.7)	2.02	1.37–2.97^††^	2.24	1.47–3.42^†^
No	4,428	301 (6.8)	Ref		Ref	

*RR crude relative risk;

**RR adjusted relative risk–adjusted for maternal BMI, Maternal age, and maternal education level in the first pregnancy;

^†^p<0.001;

^††^p<0.05.

### Recurrence risk of LBW by maternal characteristics

We performed a stratified analysis by maternal characteristics in the first birth (i.e. number of antenatal care visits, gestational age in weeks, HIV status and preeclampsia) adjusted for maternal BMI, Maternal age, and maternal education level in the first pregnancy (Table 3). The highest absolute risk of recurrence was found among mothers who delivered at <37 weeks of gestation (75.3%) and those who had preeclampsia (68.4%). The absolute risk of LBW recurrence was 35% and 29.2% among mothers with <4 antenatal care visits and those diagnosed HIV positive respectively.

**Table 3:** Recurrence risk of LBW by maternal characteristics in second pregnancy.

Risk Factor in first birth	Birth weight	Number at risk	LBW in second pregnancy			
			n	Absolute Risk	RR*	95%CI
**Antenatal Care visits**						
<4	LBW	157	55	35.0	5.00	3.58–6.98^†^
	NBW	1,559	102	6.5	Ref	
≥4	LBW	203	45	22.2	5.07	3.56–7.21^†^
	NBW	2,621	110	4.2	Ref	
**Gestational age (weeks)**						
<37	LBW	73	55	75.3	2.91	2.30–3.67^†^
	NBW	375	94	25.1	Ref	
≥37	LBW	293	48	16.4	4.55	3.21–6.43^†^
	NBW	3,862	128	3.3	Ref	
**HIV status**						
Yes	LBW	24	7	29.2	7.49	3.91–14.36^†^
	NBW	163	11	6.7	Ref	
No	LBW	264	70	26.5	5.12	3.82–6.85^†^
	NBW	3,268	160	4.9	Ref	
**Preeclampsia**						
Yes	LBW	19	13	68.4	4.37	2.60–7.35^†^
	NBW	89	16	18.0	Ref	
No	LBW	347	90	25.9	4.91	3.71–6.23^†^
	NBW	4,149	206	5.0	Ref	

^*^RR adjusted relative risk adjusted for maternal BMI, Maternal age, and maternal education in the first pregnancy;

^†^p<0.001;

^††^p<0.005.

The relative recurrence risk of LBW was higher among women with less than four antenatal care visits (RR: 5.00; 3.58–6.98), those with ≥37 weeks of gestational (RR: 4.55; 3.21–6.43), those with positive HIV status (RR:7.49: 3.91–14.36) and those without preeclampsia (RR: 4.37; 2.60–7.35) in the first pregnancy as compared to women in the reference categories (Table 3).

## Discussion

This study explored the incidence of LBW, LBW recurrence and associated factors among women who delivered singletons in subsequent pregnancies. A total of 9.5% women had LBW baby in their first pregnancy. The incidence of LBW in subsequent pregnancy was 7.1% while the absolute risk of recurrence was 28.1%. Predictors of LBW in subsequent pregnancy included delivery of LBW baby in first pregnancy, less than 4 visits attendance to antenatal care, prematurity, HIV positive status of mother and preeclampsia.

The percentage of LBW in this study is almost the same as the regional estimate (9.5%) but slightly higher than the national average (7%) which was previously reported by DHS [20]. This estimate is also the consistent to that reported among women in a community based longitudinal study in rural India [21]. Higher percentage of LBW deliveries were also reported in Tanzania, Kenya, Nigeria, Asian Countries specifically southern India and Oman as well as South America in Brazil [2, 8, 22–26]. WHO indicated that, LBW is most common in low- and middle-income countries, particularly among the most vulnerable populations [3] and without context specific interventions, these figures are rising.

Women with a previous history of LBW have been reported to have an increased risk of experiencing LBW in subsequent pregnancies [10, 13–16, 23, 24, 27]. The absolute risk of LBW recurrence in this study was also high as 28.1%, while the relative risk of over 5 times higher among women who experienced LBW in the first pregnancy compared to those who had normal birth weight. It is therefore evident that, women who experienced adverse pregnancy outcomes such as delivering LBW baby in the first or previous pregnancy are at increased risk of recurrence both in developing and developed countries [11, 13, 27]. History of LBW for both mothers and infants has also been linked with generational recurrence [13, 17].

In the present study, ANC attendance was a strong predictor of recurrences of LBW. Higher recurrence risk among women with inadequate antenatal care has also previously been reported in Pelotas, Brazil [12]. The higher risk of LBW recurrence among women with previous inadequate ANC visits could be explained by other factors e.g. preeclampsia and other complications which are common in those with inadequate ANC attendance. Although other studies did not find statistically significant association between ANC attendance and LBW recurrence [15, 27], adequate or recommended ANC attendance remains one of the important entry points where high-risk pregnancies can be early identified and monitored to prevent adverse perinatal outcomes [2, 4, 28].

Preterm birth was found to be associated with over twice the risk of LBW recurrence in this study. Similar findings were also reported elsewhere [12–14, 16, 27]. Women who were diagnosed HIV positive were also found to have 99% higher risk of recurrent LBW which is similar to findings from Zambia [15]. We also found that, the recurrence risk of LBW was more than two-fold among women with pre-eclampsia/ eclampsia in the first pregnancy as compared to those who had normotensive pregnancies. The higher recurrent LBW among preeclamptic women could be explained by the effect of preterm birth recurrence which is attributed to preeclampsia in successive pregnancies. The association between small gestational age, positive HIV status and pre-eclampsia/ eclampsia with LBW recurrence in subsequent pregnancy could probably be explained by their documented association with LBW in the first pregnancy [2, 8, 23, 24].

Although previous studies have establish the association between the recurrence risk of LBW by maternal demographic characteristics such as age, BMI, smoking status and parity [12, 15, 27, 29], these factors were considered as potential confounders in the present study.

## Conclusion

Incidence of LBW in subsequent pregnancy was 7.1% and the absolute risk of LBW recurrence in this population was 28.1% which is higher compared to 5.2% among those who delivered a normal birth weight baby. Recurrent LBW was associated with the history of LBW in first pregnancy, history of pre-eclampsia/ eclampsia, prematurity, inadequate antenatal care visits and HIV positive status of the mother. Identification of factors associated with LBW recurrence, proper post-partum care management to ensure Healthy Timing and Spacing of Pregnancy, Pre-conception care and close clinical follow-up during subsequent pregnancy may help reduce LBW recurrence.
